# Impact on Diet Quality and Burden of Care in Sapropterin Dihydrochloride Use in Children with Phenylketonuria: A 6 Month Follow-Up Report

**DOI:** 10.3390/nu15163603

**Published:** 2023-08-17

**Authors:** Maria Inês Gama, Anne Daly, Catherine Ashmore, Sharon Evans, André Moreira-Rosário, Júlio César Rocha, Anita MacDonald

**Affiliations:** 1 Birmingham Children’s Hospital, Steelhouse Lane, Birmingham B4 6NH, UK; inesrgama@gmail.com (M.I.G.); a.daly3@nhs.net (A.D.); catherine.ashmore@nhs.net (C.A.); sharon.morris6@nhs.net (S.E.); 2 Nutrition and Metabolism, NOVA Medical School, Faculdade de Ciências Médicas da Universidade NOVA de Lisboa, Campo dos Mártires da Pátria 130, 1169-056 Lisboa, Portugal; andre.rosario@nms.unl.pt (A.M.-R.); rochajc@nms.unl.pt (J.C.R.); 3 CINTESIS@RISE, Nutrition and Metabolism, NOVA Medical School, Faculdade de Ciências Médicas, NMS, FCM, Universidade NOVA de Lisboa, 1169-056 Lisboa, Portugal; 4 Reference Centre of Inherited Metabolic Diseases, Centro Hospitalar Universitário de Lisboa Central, 1169-045 Lisboa, Portugal

**Keywords:** sapropterin, food patterns, food literacy, burden of care, phenylketonuria

## Abstract

Introduction: In phenylketonuria (PKU) changes in dietary patterns and behaviors in sapropterin-responsive populations have not been widely reported. We aimed to assess changes in food quality, mental health and burden of care in a paediatric PKU sapropterin-responsive cohort. Methods: In an observational, longitudinal study, patient questionnaires on food frequency, neophobia, anxiety and depression, impact on family and burden of care were applied at baseline, 3 and 6-months post successful sapropterin-responsiveness testing (defined as a 30% reduction in blood phenylalanine levels). Results: 17 children (10.8 ± 4.2 years) completed 6-months follow-up. Patients body mass index (BMI) z-scores remained unchanged after sapropterin initiation. Blood phenylalanine was stable. Natural protein increased (*p* < 0.001) and protein substitute intake decreased (*p* = 0.002). There were increases in regular cow’s milk (*p* = 0.001), meat/fish, eggs (*p* = 0.005), bread (*p* = 0.01) and pasta (*p* = 0.011) intakes but special low-protein foods intake decreased. Anxiety (*p* = 0.016) and depression (*p* = 0.022) decreased in caregivers. The impact-on-family, familial-social impact (*p* = 0.002) and personal strain (*p* = 0.001) lessened. After sapropterin, caregivers spent less time on PKU tasks, the majority ate meals outside the home more regularly and fewer caregivers had to deny food choices to their children. Conclusion: There were significant positive changes in food patterns, behaviors and burden of care in children with PKU and their families after 6-months on sapropterin treatment.

## 1. Introduction

Phenylketonuria (PKU) is characterized by the total or partial inability to convert the amino acid phenylalanine (Phe) into tyrosine (Tyr), resulting in the accumulation of Phe in the blood and brain, leading to neurotoxicity. Lifelong treatment primarily consists of a Phe restricted diet, and excludes foods high in protein such as meat, fish, eggs, milk and dairy, nuts, pulses, flour and pasta [1]. It is supplemented with a protein substitute, either based on Phe-free amino acids and/or low Phe casein-glycomacropeptide (usually containing vitamins, minerals and long chain fatty acids), and special low protein foods (SLPF) such as bread and pasta [1].

Although dietary management is the standard treatment, adjuvant pharmaceutical treatments aim to improve metabolic control and ease Phe restriction. Sapropterin dihydrochloride (sapropterin) is a drug treatment that acts as a synthetic cofactor for phenylalanine hydroxylase (PAH). By enhancing the activity of any residual PAH, it facilitates the oxidative metabolism of Phe, aiming to lower or maintain blood Phe concentrations within target therapeutic range and/or increase natural protein or Phe tolerance [2]. Only around 30% of patients, usually with milder forms of PKU, are sapropterin responsive [2]. Long term sapropterin responsiveness is defined as a 100% increase in natural protein/Phe tolerance or maintenance of >75% blood Phe levels within target range [3].

For patients on dietary treatment only, blood Phe control usually deteriorates with age [4,5,6]. Acceptable control is increasingly harder to achieve in patients with classical PKU, who usually tolerate less than 10 g protein/day [1]. Patients with poor dietary adherence have more nutrient imbalances when compared to adherent patients [7]. Altered metabolic markers are reported in individuals with PKU, namely in carbohydrate [8], lipid [9,10] and protein [11] metabolism. There is evidence that dietary treatment of PKU may impact on several aspects of daily lives, particularly stress levels, quality of life, anxiety and depression [12,13]. A recent systematic review on the quality of life showed that seven of eight studies presented a negative correlation between PKU and quality of life in at least one domain [14]. Becsi reported that most parameters related to quality of life were within normal ranges. However, there was a considerable negative impact on food enjoyment was noted in adolescents, and emotional impact of PKU management was classified as moderate for both adolescents and caregivers [15]. A survey conducted by Ford et al. showed that 36% of adults had feelings of guilt and self-blame about PKU; 34% of adults and 17% of children had difficulties in relationships with family/partner and friends and 75% of caregivers considered PKU to be a considerable source of worry and strain [16]. An American survey also highlighted that over half (51.7%) of 625 patients reported difficulties in managing their treatment [17].

Several studies conducted on sapropterin responsive cohorts, examining blood Phe control and nutritional status, have reported an increase in natural protein intake [18,19,20,21]. It is commonly suggested that sapropterin treatment, by reducing dietary restrictions, may improve nutritional quality of the diet and promote growth. In parallel, it is expected to improve the quality of life and lessen the burden of care; this, in turn, is expected to have benefits on the mental health of patients and their caregivers [22]. However, very few studies have focused on the type and quality of protein given [23,24]. Additionally, there is a concern that with sapropterin treatment, discontinuing the protein substitute prescription may potentially leave patients at risk of nutritional imbalance, particularly if the quality of the natural protein intake is poor [25]. There is evidence from two systematic reviews that sapropterin treated cohorts have a higher BMI when compared to non-sapropterin treated groups [22,26]. However, there is very little information about how sapropterin may alter mental health status for patients or caregivers or its impact on the burden of care.

Therefore, it is essential to assess the impact of sapropterin treatment on nutritional status, quality of diet, burden of care and mental health of both patients and caregivers. In this controlled, prospective, longitudinal study, we report a 6-month follow-up of a group of sapropterin responsive children with PKU after sapropterin introduction.

## 2. Materials and Methods

### 2.1. Study Design

**Figure 1:**
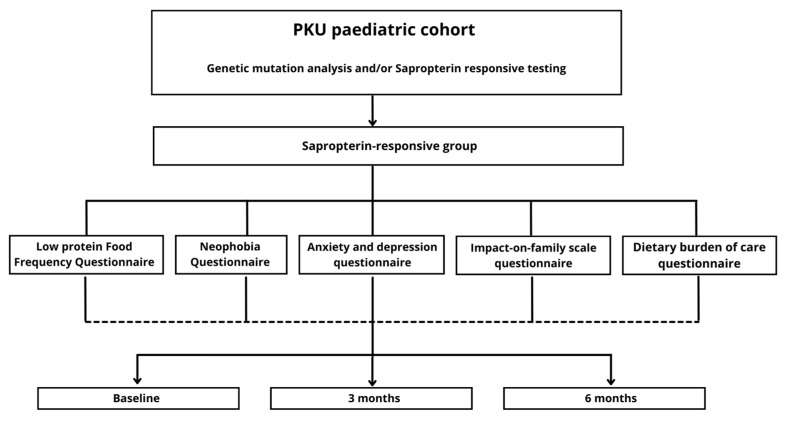
“Impact on Diet Quality and Dietary Burden of Sapropterin Dihydrochloride use in children with Phenylketonuria: A 6-months follow-up” study design.

This was an observational, controlled, longitudinal and prospective study. Its design is illustrated in Figure 1.

Study participants were children aged between 4 and 17 years and were recruited from a single centre, at Birmingham Children’s Hospital (BCH), UK. 

Inclusion criteria included children detected by newborn screening with either PAH or dihydropteridine reductase (DHPR) mutations, aged from 3 to 17 years, treated with a Phe restricted diet and had a successful sapropterin responsive test (defined as a 30% reduction in blood Phe levels).

Exclusion criteria included patients with late diagnosis of PKU, non-PKU health related conditions that may have influenced eating patterns (e.g., diabetes) and teenage pregnancy.

The caregivers of sapropterin responsive patients completed the following set of questionnaires at baseline, 3 and 6 months after the intervention: 

**Food Frequency (FFQ) questionnaire:** an 89-item validated FFQ developed for PKU was used to assess food choices and standard portion sizes [27]. Caregivers/participants were provided with a photographic portion sizes book to assist them in determining food portion size. Additionally, meal frequency, type, timing and dose of protein substitute, in addition to the number of meals eaten outside the home, were documented.

**Food and general neophobia questionnaire:** a validated questionnaire, comprising a 9-item food neophobia scale and 5 item general neophobia scale with a 7-item response (ranging from ‘always’ to ‘never’) [28]. 

**Hospital Anxiety and Depression Score (HADS) questionnaire:** a validated questionnaire to assess anxiety and depression [29]. It consisted of 7 statements about anxiety and 7 statements about depression. Respondents were given a choice of 3 answers on a scale of 0 (lower level) to 3 (higher level). This questionnaire was completed by the main caregiver and patients aged 12 years and over. 

**Impact-on-family scale questionnaire:** this validated questionnaire consisted of 24 statements [30]. The questionnaire was divided in 4 subscales: financial impact, familial-social impact, personal strain and mastery (family coping strategies). A score of 1 (higher impact) to 4 (lower impact) was recorded and calculated for each section.

**Dietary burden of care questionnaire:** a non-validated questionnaire consisting of 16 open-answer questions about daily PKU management. It was completed by caregivers only. Patients were invited to give their opinion when appropriate.

Clinical Data Collection: Demographic data (age, gender), any relevant medical history and PAH genetic mutations were extracted from the patient’s clinical records. Anthropometric measurements (weight (kg), height (cm) were obtained using SECA^®^ (SECA Medical Measuring systems, Hamburg, Germany) scales (model 875) (with light clothes and without shoes) (measured to the nearest 0.1 kg) and stadiometer (model 213) (measured to the nearest 0.1 cm). Body mass index (BMI) (kg/m^2^) z-scores were calculated using hospital software and classified according to the World Health Organization (WHO) criteria [31]. 

Routine blood Phe levels were prospectively collected during the 6 months follow-up period. Trained caregivers collected weekly morning fasting blood spots on filter cards, (Perkin Elmer 226, Standard NBS, Public Health England, London, UK). All parents/caregivers had received prior training on blood spot collection and their technique was reviewed prior to sapropterin introduction as recommended by the British Inherited Metabolic Diseases Group (BIMDG) protocol. Blood spots were returned to the laboratory at Birmingham Children’s Hospital for analysis. All the cards had a standard thickness, and the blood Phe concentrations were calculated on a 3.2 mm punch by MS/MS tandem mass spectrometry. Retrospective blood Phe concentrations were collected from June 2021 to December 2021, and the mean blood Phe concentrations was estimated as the baseline mean.

Ethical approval for this study protocol was granted by the UK Wales REC 6 independent NHS Research Ethics Committee (REC reference: 22/WA/0143; IRAS ID: 314071). The study was conducted in accordance with the ethical principles expressed in the Declaration of Helsinki, English law and Good Clinical Practice guidelines. Caregivers gave informed consent and children assent if of an appropriate age and understanding. 

### 2.2. Statistical Analysis

Descriptive statistics were presented as numbers and percentages for categorical variables, as the mean and standard deviation (SD) for continuous variables or as the median and interquartile range if the variable empirical distribution function was skewed. Results are summarised as descriptive statistics for baseline, 3 and 6 months. Parametric tests and nonparametric tests based on normality assumptions were used to compare groups. The Shapiro–Wilks test examined normality assumptions of the variable distributions. Multiple comparisons between groups were conducted using the Friedman test when testing hypotheses about variables. When statistical differences were detected, the pairwise comparison adjusted to Bonferroni was performed. Differences were considered statistically significant when *p* < 0.05. Statistical analysis was performed using statistical SPSS Statistics version 27.

## 3. Results

### 3.1. Characterization of Study Cohort

Seventeen children with PKU, aged 10.8 ± 4.2 years, were identified as sapropterin responsive (defined as a mean 30% reduction in blood Phe after 28 days of treatment with sapropterin at 20 mg/kg) from January to July 2022, and were followed up for 6 months. Patients’ baseline characteristics are described in Table 1.

Using the BIOPKU database for genotype-specific BH4-responsiveness [32], five patients were classified as classical (cPKU), eight as moderate (mPKU), two as mild PKU (HPA) and two patients had DHPR deficiency. In two patients, the nucleotide aberrations or mutations were not described/were unknown, so phenotype was established based on diagnostic blood Phe values and Phe tolerance prior to sapropterin testing. Two adolescent girls (aged 15 and 16 years) discontinued sapropterin treatment during the study period and subsequently resumed dietary treatment only. In one girl, sapropterin was stopped due to non-adherence with treatment while the other chose to discontinue treatment due to stress-related issues after 3-months on the drug. Both patients agreed to further follow up as part of this study.

### 3.2. Metabolic Control

Over the 6-month follow up period, blood Phe levels were within target range for all three time-points for this cohort of responsive patients (Table 2). The two patients who discontinued treatment were excluded from the blood Phe analysis after sapropterin was discontinued. 

### 3.3. Total Protein Intake

Natural protein intake increased significantly, from 11 g/day at baseline to 25 g/day at 3-months (*p* = 0.001) and 30 g/day at 6-months (*p* < 0.0001) (Table 3). Although there were statistical differences between protein equivalent from protein substitute intake during the study, these were not significant between any time-point (0–3 m; 3–6 m; 0–6 m). The median protein equivalent intake from protein substitute intake remained similar at baseline and at 6-months.

### 3.4. Anthropometry

For children aged ≤ 10 years, there was a significant increase in weight z-score (Table 4), but not mean BMI z-score. In contrast, there were no significant differences for children/adolescents aged > 10 years in weight, height and BMI z-scores throughout the study.

### 3.5. Food Frequency Questionnaire

During the 6-month study follow-up period, there were changes in food patterns for protein-containing food groups, such as cow’s milk, meat, fish, eggs, regular bread and pasta. These changes were measured by the number of food/drink portions consumed each week (Table 5). Between baseline and 6-months, there was a significant increase in regular bread (*p* = 0.014) and regular pasta (*p* = 0.031) and a decrease in low protein pasta (*p* = 0.002) intake. Additionally, between baseline and 3-months, patients significantly decreased their intake of low protein milk (*p* = 0.018) and low protein pasta (*p* = 0.003).

### 3.6. Neophobia Questionnaire

Throughout the study period, no significant differences were observed in food neophobia and general neophobia scores (Table 6). 

### 3.7. HADS Questionnaire

Over the 6 months, for the overall group (both caregivers and adolescents aged ≥12 years), there was a tendency for a decrease in anxiety scores (*p* = 0.051), but no significant change was observed in depression. However, when analyzing caregivers separately, there were statistically significant lower scores for both anxiety and depression (Table 7). Lower scores on the sum of anxiety and depression scales within this questionnaire were interpreted as indicating less anxiety and depression, while higher scores were indicative of more anxiety and depression. 

### 3.8. Impact on Family

During the 6-month study period, there was a significant decrease in familial-social impact and personal strain (Table 8). Lower sub-scores translated to higher impact on the family, while higher sub-scores were associated with lower impact on the family.

### 3.9. Caregivers Burden of Care Questionnaire

Thematic analysis was conducted about the hours spent on PKU care by caregivers, food expenditure, issues with protein substitute administration, low protein food use and parental/child disagreements concerning food choices. 

Table 9 describes the number of hours caregivers spent caring for PKU related issues, including meal planning, preparing food, cooking, shopping, liaising with delivery companies and blood testing. There was a decrease in the time spent on these activities over 6 months, mainly associated with less time preparing and cooking lower protein meals and the ability of the child to eat the same food as the rest of the family. Some parents reported spending more money on food with sapropterin treatment, which was mostly associated with trying new foods (and food wastage if foods were disliked), purchase of gluten free foods instead of using low protein prescription foods and eating more meals outside the home. Most parents were happy that their children had more enjoyable food experiences. Verbatim comments from caregivers or teenagers included:

- *“We spend less time in shops. I’m not constantly looking at packets when we go shopping anymore.”*
- *“Being able to have more convenience with foods readily available in the supermarket it’s much easier.”*
- *“Expenditure increased because now he can have more gluten-free foods.”*

At baseline, all patients used SLPF’s, particularly bread (59%, *n* = 10), pasta (88%, *n* = 15) and milk (82%, *n* = 14). These were still the most common SLPF’S at 6-months, but less were consumed, and some children (*n* = 2) were able to discontinue all SLPF. Verbatim comments by caregivers were:

- *“We don’t use low protein foods anymore.”*
- *“He has the same rice and pasta as his brother now.”*

During the 6-month study, families increased the number of times they ate out in restaurants or cafes. At 6-months, most families (76%, *n* = 13) ate out at least once a week, whereas at baseline this was only 24% (*n* = 4). Results are shown in Table 10. At baseline, caregivers described there was little pleasure in eating out, particularly as they had many disagreements with their children about food choices and portion sizes. Overall, there was a decrease in the number of times caregivers had to deny food choices to their children. Caregivers reported:

- *“I don’t have to tell our life story to explain why he can’t eat something; he is just having a smaller portion than us.”*
- *“We went camping with no electricity. It would absolutely not happen before the drug. It was brilliant to see him just enjoy it as everybody else.”*
- *“It helped me not having to tell him no all the time.”*

## 4. Discussion

This prospective study in a cohort of sapropterin responsive children with PKU reports its impact on family and associated burden of care. It also describes changes in food patterns, nutritional status and its effect on family mental health. Natural protein intake at least doubled after 6-months of sapropterin treatment without any loss of metabolic control. There was a decreased dependence on the use of SLPFs. Anxiety and care burden lessened for parents with an increase in social interaction involving food (e.g., parties, meals outside the home) for children with PKU. 

The individual patient benefits from an increase in natural protein were dependent on their original protein tolerance. With sapropterin, most children could tolerate over 20 g/day of natural protein, while a minority with mild PKU tolerated over 40 g/day. When children achieved a protein tolerance of 20 g/day, they were encouraged to include eggs, cheese and even small portions of meat and fish in their diet. If their tolerance remained less than 20 g/day, their protein sources were limited to milk, yoghurt, cereals, vegetables and other plant proteins. Ideally, when natural protein intake is increased, high biological quality protein should be introduced. However, new food choices may be refused by patients, or their protein tolerance may not be high enough to permit the inclusion of such foods. Even when protein exceeded 20 g/day, there was an immediate patient resistance to expanding the range of foods eaten. Children expressed uncertainty about the taste, texture and smell of meat and fish. They described meat as ‘too chewy.’ In contrast, regular bread and pasta were easily accepted, and children were happy to discontinue eating SLPF. Similar results were reported by Thiele et al. [23,24], in a group of sapropterin treated patients, where intake of SLPF significantly decreased in favor of higher natural protein food sources. This change may considerably improve the quality of nutritional intake [33].

Any new food patterns are conditioned by the extent of dietary protein liberalization [34]. To aid this process, a UK dietetic consensus was developed to help health professionals guide patient food choice when increasing protein allowance [34]. We observed that dietary changes that sustained good food choices occurred over a long time [35]. Overall, changes in food patterns were a dynamic and complex process. It was recognized that it was important to counsel the parents and patients regularly and manage their expectations. Initially, families made rapid increases in their children’s protein intake. The BIMDG/NHS sapropterin guidance had to be followed in England. The protocol stated that the drug had to be discontinued after 6-months if patients did not double their natural protein intake. This created an urgency to increase natural protein intake but arguably this did not allow adequate time to develop healthy eating practices. Caregivers chose protein containing foods that they knew their children would eat rather than selecting healthy foods that may be initially rejected and would take time to establish acceptance. For a quick change, many families replaced SLPFs with regular varieties of the same foods, such as bread and pasta. Additionally, the uncertainty about the final natural protein tolerance prompted professionals and caregivers to exercise caution when selecting the type of foods, particularly if the drug treatment was withdrawn at 6 months. Furthermore, the age of the children influenced the food sources of protein offered by caregivers. They were reluctant to introduce meat and fish to young children due to protein allowance restrictions. As a result, the portion sizes of permitted foods might not change with increasing age of the child [34].

Only modest reductions were made in reducing protein substitute prescriptions. This was primarily associated with the quality of the natural protein eaten. Protein substitute is usually supplemented with vitamins and minerals, and it provides an important nutritional safety net in PKU. After 6 months of sapropterin treatment, the quality of the nutritional intake from food was inadequate and common foods eaten were low in nutrients such as calcium, iron and zinc. Consequently, and following 6 months of sapropterin treatment, children were given individual dietary goals defining a minimum daily intake of higher protein foods such as cheese and eggs that should be eaten before protein equivalent from protein substitute could be reduced. Additionally, it is well established that Phe-free protein equivalent from protein substitute helps lower blood Phe concentrations [36,37,38,39]. This plays a crucial role in maintaining blood Phe concentrations within therapeutic target levels, specifically during illness. Consequently, it may be challenging to substantially decrease or stop protein substitute dosage in some patients. 

Our results showed no differences in BMI z-scores over 6 months, which is consistent with findings from other cohorts of sapropterin treated patients [18,21,40,41]. However, two systematic reviews have shown that there was a trend for higher BMI in sapropterin treated patients [22,26]. The reasons for the increased BMI are unknown, but authors suggest that these changes could be associated with unhealthy food choices after an increase of natural protein in the diet. Therefore, it is imperative to closely monitor patients following any relaxation of protein intake and a change in dietary patterns with regular, systematic monitoring of nutritional intake and body composition status. 

We found that both anxiety and depression levels were significantly lower for caregivers when their children were taking sapropterin but not in patients. The high level of caregiver distress prior to sapropterin commencement was similar to other studies [42,43,44,45]. Caregivers of children treated with sapropterin were more relaxed, less fearful and more positive. Notably, one parent discontinued anti-depressant usage following sapropterin introduction. For teenagers, there was no change in anxiety or depression scores, but it is important to acknowledge that the number of patients studied was small. 

Overall, caregivers reported a significant alleviation with both the familial-social impact and personal strain sub-scales in the impact on family questionnaire. This questionnaire was adapted for our PKU population and aimed to assess the impact of a child’s PKU within the family environment. The questionnaire comprised four categories, spanning financial aspects, social impact of PKU within the family, personal strain on the caregiver and strategies used to manage the condition. The familial-social impact and personal strain sub-scales were composed of statements related to social interactions within or outside the family nucleus (e.g., “We have to change plans at the last minute because of PKU”; “We are unable to travel”) and with the direct impact on the caregiver (e.g., “Nobody understands the burden I carry”; “It’s hard to find a reliable person to take care of my child”), respectively. This questionnaire served as an objective measure to assess burden of care objectively with an additional complementary questionnaire. Similar results were also reported from the burden of care questionnaire, where introduction of sapropterin treatment increased family social interactions such as eating out, holidays and attending parties. Parents also reported they spent fewer hours on PKU related tasks, felt more relaxed, were less worried about their child’s treatment and felt less guilty about having to deny food choices to their children. There were fewer arguments about food choices so family life was more pleasant. Similar results were reported by a recent Dutch study that showed that caregivers of children with PKU on sapropterin treatment were less limited by their child’s dietary restrictions when choosing holiday destinations [46]. 

Sapropterin has been hypothesized to decrease dietary burden and improve quality of life; however, reports from the literature show conflicting results [47,48,49,50]. It is possible that assessment tools used in other studies might not have been sensitive enough to detect more subtle changes in patients’ daily lives. The experiences of children on dietary restrictions, their caregivers and families describing the issues they experience daily have been well described but have failed to be identified through the general quality of life questionnaires [16,51,52]. 

The study did have some limitations. Although these results describe the patient’s experiences over the first 6-months of sapropterin usage, this cohort of patients are continuing their study follow up for a period of 2-years, which will allow an extended timeframe to assess long term outcome. Our sample size of sapropterin patients was small but this was representative of the population of patients with PKU within the clinic. Additionally, the study contained several questionnaires, and their completion was time consuming for parents and patients. However, the researchers worked with the participants to ensure the necessary information was collected in a timely manner and there was no suggestion of participant fatigue related to the number and length of questionnaires completed. The HADS and the Impact on Family questionnaires were not PKU-specific but, due to a lack of PKU specific tools, we considered these validated questionnaires were suitable to collect the necessary data. Finally, the burden of care questionnaire, a non-validated questionnaire developed by the study researchers, measured the issues we identified as being of importance to the families. Further validated tools should be developed to evaluate the burden of care in PKU.

## 5. Conclusions

Sapropterin dihydrochloride for the treatment of PKU has been available worldwide for more than a decade, and although many other new treatment advances are in the pipeline, studies on pharmaceutical treatments and their impact on dietary management and burden of care are still scarce. We were able to demonstrate, for the first time, several complex and multi-factored changes in dietary patterns and care burden when the treatment modality changed. More research is needed on long-term diet quality, patient and caregiver mental health and burden of care when introducing new treatments in PKU. Appropriate patient education, support and adequate time duration is essential when introducing dietary modifications to guarantee a smooth and nutritionally adequate transition to a liberalized diet in sapropterin-responsive cohorts, enabling a long-term healthier status. 

In PKU, dietitians, health care professionals and national health systems should work together to improve patient access to new therapies and develop treatment pathways that advance treatment practice and lead to improved and sustained patient health.

## Figures and Tables

**Table 1:** Patients baseline characteristics.

Sapropterin-Responsive Patients (*n* = 17)		
Male (*n*, %)	11 (65%)	
Female (*n*, %)	6 (35%)	
Age (mean ± SD)	10.8 ± 4.2	
<6 years	*n* = 2	
6–12 years	*n* = 10	
>12 years	*n* = 5	
Genetic mutations (*n*, %)		
Classical PKU (cPKU)	5 (29%)	
Moderate PKU (mPKU)	8 (47%)	
Mild PKU (HPA)	2 (12%)	
DHPR Deficiency	2 (12%)	
Sapropterin dosing (median [P_25_–P_75_])		
Sapropterin (mg/kg)	20 [10–20]	
Blood Phe control (median [P_25_–P_75_])		
Blood Phe (µmol/L)	258 [231–398]	
Natural protein and protein equivalent intake (median [P_25_–P_75_])		
Natural Protein (g/day)	11 [9–14]	
Protein equivalent from protein substitute (g/day)	60 [57–60]	
Anthropometrics (mean ± SD)		
	≤10 years old (*n* = 9)	>10 years old (*n* = 8)
Weight z-score	0.87 ± 1.55	0.49 ± 1.36
Height z-score	0.37 ± 0.69	0.05 ± 0.96
BMI z-score	0.88 ± 1.76	0.57 ± 1.19

(*n*, %; Mean ± SD; Median [P25–P75]).

**Table 2:** Changes in blood Phe at baseline, 3- months and 6-months.

Metabolic Control (*n*= 17) (Median [P_25_–P_75_])				
	Baseline	3-Months Post Sapropterin	6-Months Post Sapropterin	Friedman Test (*p*-Value < 0.05)
Mean blood Phe (µmol/L)	258 [231–398]	310 [277–356]	263 [232–359]	0.731

**Table 3:** Changes in natural protein at baseline, 3- months and 6-months.

Natural Protein and Protein Equivalent Intake (*n* = 17) (Median [P_25_–P_75_])				
	Baseline	3-Months Post Sapropterin	6-Months Post Sapropterin	Friedman Test (*p* Value < 0.05)
Natural protein (g/day)	11 [9–14]	25 [15–34]	30 [23–38]	<0.001
Protein equivalent (g/day) from protein substitute	60 [57–60]	55 [39–60]	60 [40–60]	0.002

**Table 4:** Changes in anthropometrics at baseline, 3- months and 6-months.

Anthropometrics (*n* = 17) (Mean ± SD)				
Study Follow Up Duration	Baseline	3-Months Post Sapropterin	6-Months Post Sapropterin	Friedman Test (*p* Value < 0.05)
≤10 years old *n* = 9				
Weight z-score	0.87 ± 1.55	1.00 ± 1.66	1.07 ± 1.67	0.045
Height z-score	0.37 ± 0.69	0.56 ± 0.72	0.52 ± 0.85	0.124
BMI z-score	0.88 ± 1.76	0.82 ± 1.94	1.06 ± 1.82	0.121
>10 years old *n* = 8				
Weight z-score	0.49 ± 1.36	0.49 ± 1.29	0.59 ± 1.31	0.417
Height z-score	0.05 ± 0.96	0.18 ± 1.02	0.28 ± 1.21	0.197
BMI z-score	0.57 ± 1.19	0.56 ± 1.20	0.57 ± 1.06	0.798

**Table 5:** Changes in food frequency (FFQ) at baseline, 3-months and 6-months.

FFQ (Portions per Week) (*n* = 17) (Median [P_25_–P_75_])					
Food	Portion Size	Baseline	3-Months Post Sapropterin	6-Months Post Sapropterin	Friedman Test (*p* Value <0.05)
Cow’s milk	30 mL	0 [0–1.5]	1 [0–66]	1 [0–42]	0.001
Low protein milk	200–250 mL	7 [4.5–14]	3.5 [1–11]	5 [0–7]	0.007
Regular cheese	15–20 g	0 [0–0]	0 [0–3]	0 [0–0]	0.165
Low protein cheese	20 g	3.5 [1–5]	0 [0–3]	0 [0–3]	0.172
Meat/fish/eggs	60–80 g 1 medium egg	0 [0–0.5]	1 [0–6]	0 [0–7]	0.005
Low protein meat/fish/eggs	80 g	0 [0–1.5]	0 [0–0]	0 [0–1]	0.094
Regular bread	30–70 g	0.5 [0–5.5]	7 [4.5–12]	9 [6–14]	0.010
Low protein bread	30–70 g	1.5 [0–6.5]	0 [0–1]	0 [0–2]	0.028
Regular pasta	15 g (cooked)	0 [0–2]	2 [0–5.5]	3 [0–13]	0.011
Low protein pasta	80–100 g (cooked)	2.5 [2–5]	0 [0–2]	0 [0–2]	<0.001
Chips and processed potatoes	30–55 g	9 [3–14]	11.5 [3.5–16]	6 [6–13]	0.678
Vegetables	20–60 g	8 [5–17]	12 [4.5–15.5]	8 [4–19]	0.854
Fruit (fresh)	80–100 g (1 piece)	6 [0.5–14]	3 [0.5–10.5]	7 [3–14]	0.614

**Table 6:** Changes in neophobia scores at baseline, 3- months and 6-months.

Neophobia Questionnaire (*n* = 17) (Mean ± SD)				
	Baseline	3-Months Post Sapropterin	6-Months Post Sapropterin	Friedman Test (*p* Value < 0.05)
Food neophobia	35.94 ± 6.15	35.69 ± 4.71	33.65 ± 4.12	0.201
General neophobia	16.47 ± 8.73	17.56 ± 8.41	20.53 ± 7.23	0.448

**Table 7:** Changes in anxiety and depression (HADS) at baseline, 3- months and 6-months.

HADS Questionnaire (*n* = 17) (Median [P_25_–P_75_]; Mean ± SD)				
	Baseline	3-Months Post Sapropterin	6-Months Post Sapropterin	Friedman Test (*p* Value <0.05)
Anxiety score (overall) (caregivers and adolescents ≥ 12 years old)	8 [5–16]	8.5 [5–14]	6 [5–13]	0.051
Depression score (overall) (caregivers and adolescents ≥ 12 years old)	6 [2–7]	3.5 [2–6.5]	4 [1–7]	0.336
Anxiety score (caregivers)	10.1 ± 5.5	8.3 ± 5.2	6.8 ± 4.9	0.016
Depression score (caregivers)	5.2 ± 3.6	3.8 ± 3.0	3.8 ± 3.1	0.022
Anxiety score (adolescents ≥ 12 years old)	9.8 ± 6.6	10.8 ± 7.5	9.8 ± 7.9	0.936
Depression score (adolescents ≥ 12 years old)	4.8 ± 3.1	5.5 ± 4.0	5.4 ± 5.3	0.424

Normal = 0–7; Borderline abnormal = 8–10; Abnormal = 11–21.

**Table 8:** Changes in impact on family at baseline, 3- months and 6-months.

Impact on Family Scale Questionnaire (*n* = 17) (Median [P_25_–P_75_])				
	Baseline	3-Months Post Sapropterin	6-Months Post Sapropterin	Friedman Test (*p* Value < 0.05)
Financial Impact	7 [6–9]	8 [5–11]	11 [7–11]	0.062
Familial-Social Impact	15 [11–19]	19 [15–22]	21 [16–24]	0.002
Personal Strain	12 [9–15]	15 [9–17]	17 [11–20]	0.001
Mastery	8 [8–9]	8 [6–10]	7 [7–9]	0.861

**Table 9:** Caregivers reports on amount of time and food expenditure related to PKU care at baseline, 3-months and 6-months post sapropterin-testing.

	Baseline	3-Months Post Sapropterin	6-Months Post Sapropterin
Hours spent on PKU management chores	All day: 35% (*n* = 6)	More time spent on PKU care	
		29% (*n* = 5)	6% (*n* = 1)
	≤42 h/week: 12% (*n* = 2)		
		Less time spent on PKU care	
	≤28 h/week: 35% (*n* = 6)	35% (*n* = 6)	53% (*n* = 9)
	≤14 h/week: 18% (*n* = 3)		
		Same time spent on PKU care	
		35% (*n* = 6)	41% (*n* = 7)
Food Expenditure		More money spent on food	
		53% (*n* = 9)	59% (*n* = 10)
		Less money spent on food	
		18% (*n* = 3)	29% (*n* = 5)
		Same money spent on food	
		29% (*n* = 5)	12% (*n* = 2)

**Table 10:** Caregivers report on the number of times eating out and on denying food choices to their children, at baseline, 3-months and 6-months post sapropterin-testing.

	Baseline	3-Months	6-Months
Times eating out	<1x/month		
	59% (*n* = 10)	35% (*n* = 6)	24% (*n* = 4)
	1–3x/month		
	18% (*n* = 3)	29% (*n* = 5)	0% (*n* = 0)
	≥1 a week		
	24% (*n* = 4)	35% (*n* = 6)	76% (*n* = 13)
Deny food choices	1–2x/week		
	35% (*n* = 6)	41% (*n* = 7)	59% (*n* = 10)
	3–5x/week		
	24% (*n* = 4)	35% (*n* = 6)	18% (*n* = 3)
	All day		
	41% (*n* = 7)	24% (*n* = 4)	24% (*n* = *4*)

## Data Availability

Not applicable.
